# Data Quality Assessment on Congenital Anomalies in Ontario, Canada

**DOI:** 10.3389/fped.2020.573090

**Published:** 2020-11-20

**Authors:** Qun Miao, Aideen M. Moore, Shelley D. Dougan

**Affiliations:** ^1^The Better Outcomes Registry & Network (BORN) Ontario, Ottawa, ON, Canada; ^2^Children's Hospital of Eastern Ontario (CHEO) Research Institute, Ottawa, ON, Canada; ^3^School of Epidemiology and Public Health, University of Ottawa, Ottawa, ON, Canada; ^4^The Hospital for Sick Children, Toronto, ON, Canada; ^5^The Hospital for Sick Children Research Institute, Toronto, ON, Canada; ^6^Department of Paediatrics, University of Toronto, Toronto, ON, Canada

**Keywords:** the BORN information system (BIS), congenital anomalies, the Canadian institute for health information discharge abstract database (CIHI-DAD), data quality, agreement, kappa test

## Abstract

**Background:** Congenital anomalies (CAs) are a major cause of infant morbidity and mortality in Canada. Reliably identifying CAs is essential for CA surveillance and research. The main objective of this study was to assess the agreement of eight sentinel anomalies including: neural tube defects (NTD), orofacial clefts, limb deficiency defects (LDD), Down syndrome (DS), tetralogy of Fallot (TOF), gastroschisis (GS), hypoplastic left heart syndrome (HLHS) and transposition of great vessels (TGA) captured in the BORN Information System (BIS) database and the Canadian Institute for Health Information (CIHI) Discharge Abstract Database (DAD).

**Methods:** Live birth and stillbirth records between the BIS and CIHI-DAD in the fiscal years of 2012–2013 to 2015–2016 were linked using 10 digit infant Ontario Health Insurance Plan (OHIP) numbers. Percent agreement and Kappa statistics were performed to assess the reliability (agreement) of CAs identified in the linked BIS and CIHI-DAD birth records. Then, further investigations were conducted on those CA cases identified in the CIHI-DAD only.

**Results:** Kappa coefficients of the eight selected CAs between BIS (“Confirmed” or “Suspected” cases) and CIHI-DAD were 0.96 (95% CI: 0.93–0.98) for GS; 0.81 (95% CI: 0.78–0.83) for Orofacial clefts; 0.75 (95% CI: 0.72–0.77) for DS; 0.71 (95% CI: 0.65–0.77) for TOF; 0.62 (95% CI: 0.55–0.68) for TGA; 0.59 (95% CI: 0.49–0.68) for HLHS, 0.53 (95% CI: 0.46–0.60) for NTD-all; and 0.30 (95% CI: 0.23–0.37) for LDD.

**Conclusions:** The degree of agreement varied among sentinel CAs identified between the BIS and CIHI. The potential reasons for discrepancies include incompleteness of capturing CAs using existing picklist values, especially for certain sub-types, incomplete neonatal special care data in the BIS, and differences between clinical diagnosis in the BIS and ICD-10-CA classification in the DAD. A future data abstraction study will be conducted to investigate the potential reasons for discrepancies of CA capture between two databases. This project helps quantify the quality of CA data collection in the BIS, enhances understanding of CA prevalence in Ontario and provides direction for future data quality improvement activities.

## Background

Congenital anomalies (CAs) are a major cause of infant morbidity and mortality in Canada. The overall prevalence rate of anomalies has been estimated as 3.9–4.5% among all births between 1998 and 2009 in Canada (1, 2). CAs have a significant impact on the affected children, their families, and the health care system in Canada (1).

In 1966, the Public Health Agency of Canada (PHAC) established the Canadian Congenital Anomalies Surveillance System (CCASS) to allow passive surveillance of anomaly cases and facilitate analysis and interpretation of Canadian birth population data (1, 3). A primary source of data for congenital anomalies surveillance in Canada is hospital administrative data from the Canadian Institutes for Health Information (CIHI) (4, 5), specifically the Discharge Abstract Database (DAD) (5).

The DAD collects information on obstetrical deliveries, newborns and stillbirths from all acute inpatient hospitals in Canada, except Quebec (6). Newborn and childhood anomalies diagnoses in acute hospitals are recorded and classified according to the 10th International Statistical Classification of Diseases and Related Health Problems (ICD-10), Canadian Adaptation (CA) (6, 7). However, CIHI data has several limitations, e.g., records capturing termination of pregnancy are extremely limited, as are records outlining fetal anomalies identified in the prenatal period. Further, CIHI records do not include environmental exposures data to examine the risk factors for anomalies (4). In an effort to overcome the limitations of CIHI data and to enhance the existing surveillance system, more recently, the PHAC has worked in collaboration with the provinces and territories (PTs) to collect CA surveillance data on a PT level (4).

One example of a provincial contributor to the CCASS is the Better Outcomes Registry & Network (BORN) Ontario. BORN Ontario is a prescribed registry that collects data on every pregnancy and birth in the province (8). As the largest province in Canada (9), Ontario has close to 40% of all births nationally (~140,000 per year), of which 2.6% are home births (personal communications). All births in Ontario are captured in the BORN registry (8). Hospitals, midwifery practice groups, screening programs, laboratories and clinics across the province contribute data to the registry via the BORN Information System (BIS), which integrates data collected at the point of care across the continuum into a single maternal-child registry (8). BORN has developed rigorous methods for CA capture before birth, at the time of birth and postpartum [birth and neonatal intensive care unit (NICU)/special care nursery (SCN) encounters] (10).

Compared to the DAD, the BORN registry contains more information on maternal obstetrical history, environmental and behavior exposures and characteristics, allowing improved monitoring of anomalies and other health-related outcomes, as well as the ability to explore risk factors for congenital anomalies and evaluate the determinates of prenatal and neonatal health. In addition, fetal anomalies and prenatal screening data are collected in the BIS, which allows monitoring and examination of the status of early termination or pregnancy loss associated with congenital anomalies.

Data quality is essential for CA surveillance and research. The aim of this study is to ensure reliable data and monitor data quality continuously in the BIS (11). Since both the BIS and DAD collect similar newborn anomalies diagnosis information and birth data from acute hospitals in Ontario, we are able to compare the prevalence of anomalies detected in the BIS and DAD, as well as link the BIS and DAD data to compare the reliability of CA diagnoses in both databases (8). The objectives of this study were to: (1) describe the prevalence rates of eight sentinel anomalies including: neural tube defects (NTD), orofacial clefts, limb deficiency defects (LDD), Down syndrome (DS), tetralogy of Fallot (TOF), gastroschisis (GS), hypoplastic left heart syndrome (HLHS) and transposition of great vessels (TGA) in Ontario based on the BIS data and the CIHI-DAD separately; and (2) assess the agreement of these sentinel anomalies identified between the BIS and the CIHI-DAD.

## Methods

### Study Design and Setting of the Study

For the first objective, all hospital stillbirth (including termination at the infant/fetus's gestational age (GA) at birth ≥20 weeks or birth weight at birth ≥500 g) or live birth records of Ontario residents in the fiscal years of April 1 2012 – March 31 2013 to April 1 2015 - March 31 2016 were captured in the BIS and the CIHI-DAD separately. In the BIS, a data linkage was conducted to identify the eight selected CAs either from a prenatal stage for fetal anomalies or a postnatal stage for newborn anomalies. This internal linkage was performed to link individual records across different encounter databases where CAs might be reported (e.g., prenatal screening, antenatal specialty, birth, or NICU/SCN records). Due to data limitation, we were only able to link singletons with anomalies identified during the prenatal stage but both singletons and high order births at birth and postnatal stages. Anomalies collected in the BIS can be flagged as “Suspected” or “Confirmed” cases. The determination of “Suspected” or “Confirmed” cases depends on the type of CA and is based on clinical assessment performed at each clinic or hospital site. In general, if a CA has not been confirmed yet, the CA is entered as “suspected” in the BIS. Suspected anomalies are identified by indirect means (e.g., prenatal ultrasound). Confirmed anomalies are to be recorded through direct means (e.g., amniocentesis or direct clinical evaluation) (personal communications with clinical experts). At many sites, nurses, genetic counselors or clinic clerks are responsible for data entry by selecting values from a multi-option anomaly picklist; in other centers, birth anomalies are identified through direct upload from the hospital electronic health record. BORN registry adopts a passive case ascertainment method to collect anomaly data in BIS. We calculated both an overall rate which combined “Suspected” and “Confirmed” cases; as well as a “Confirmed” only prevalence rate in the BIS. Among the BIS, 11.3% of these eight anomaly cases were stillbirths and 8.8% of them were terminated. In the CIHI-DAD, we identified cases of the eight CAs among stillbirth and live birth records using ICD-10-CA codes starting with letter “Q” in the report list provided by the PHAC (2). We found 7.9% of these CAs cases were stillbirths in the DAD.

The provincial health card number (Ontario Health Insurance Plan [OHIP]) is a unique identifier, and has been assigned to almost all newborns (8). Therefore, for the second objective, we were able to use the 10 digit infant OHIP number to deterministically link birth records between the BIS and CIHI-DAD in the the 4-year period. Most stillbirths and a small number of livebirths records in both databases do not have an OHIP number. Therefore, we were not able to perform linkage using an OHIP number. Among the unlinked cases, the infants have one or more diagnoses of these eight anomalies. In this linked cohort, we further obtained records with anomaly diagnoses either through the BIS or the DAD. The picklist values for the 8 anomalies in the BIS and corresponding diagnosis ICD-10-CA Q codes used for identifying those eight anomalies in the DAD are listed in Table 1.

**Table 1:** Picklist values in BIS and ICD-10-CA code in CIHI-DAD for Selected Congenital Anomalies.

**Congenital anomalies**	**Picklist values in BIS**	ICD-10-CA Q code in CIHI-DAD^*^
Neural tube defects (NTD)-all	Acrania; exencephaly; Exencephaly; Craniorachischisis; Iniencephaly; Encephalocele; NTD (neural tube defect) with hydrocephalus; NTD (neural tube defect) without hydrocephaluswithout hydrocephalus	Anencephaly and similar anomalies (Q00); Spina bifida without anencephaly (Q05 if not Q00.0); Encephalocele (Q01)
Orofacial clefts	MOUTH-Cleft palate; MOUTH-Cleft lip; MOUTH-Cleft lip & palate	Cleft palate only (Q35 excluding Q35.7); Cleft lip only (Q36); Cleft lip with or without cleft palate (Q36, Q37)
Limb deficiency defects (LDD)	Generalized/other-Limb reduction defect(s) (LRD) - upper limb; Hands/feet-Clenched hands (persistently); Hands/feet-Radial ray anomaly (absent thumb); Hands/feet-Adactyly (absent fingers/ toes); Hands/feet-Ectrodactyly (lobster-claw / cleft hand); Generalized/other-Limb reduction defect(s) (LRD) - lower limb; Generalized/other-Phocomelia	Q71: Reduction defects of upper limb Q72: Reduction defects of lower limb Q73: Reduction defects of unspecified limb
Gastroschisis (GS)	Gastroschisis	Gastroschisis (Q79.3)
Down syndrome (DS)	Trisomy 21 (Down syndrome); Trisomy 21 (Down syndrome) - mosaic; Trisomy 21 (Down syndrome) - translocation	Down syndrome (Q90)
Tetralogy of Fallot (TOF)	Tetralogy of Fallot	Tetralogy of Fallot (Q21.3)
Hypoplastic left heart syndrome (HLHS)	HLHS (hypoplastic left heart syndrome)	Hypoplastic left heart syndrome (Q23.4)
Transposition of great vessels (TGA)	Double outlet ventricle (DOV); Transposition of great vessels (TGA); Transposition of great arteries - congenitally corrected (CCTGA)	Transposition of great vessels (Q20.1, Q20.3, Q20.5)

*BIS, BORN Information System; CIHI, Canadian Institute for Health Information; DAD, Discharge Abstract Database*.

* *The definition and grouping of congenital anomalies was based on the report list provided by the PHAC*.

### Analysis

The overall BIS prevalence rate of one specified CA is defined as the number of live births or stillbirths having a “confirmed” or “suspected” specified CA that was identified from the BIS, expressed as a proportion of the total number of live births and stillbirths delivered in an Ontario hospital with infant residence in Ontario. The BIS “confirmed” prevalence rate of one CA was defined as the number of live births or stillbirths identified having a “confirmed” specified CA that was identified from the BIS, expressed as a proportion of the total number of live births and stillbirths delivered in hospitals with infant residence in Ontario. The DAD prevalence rate of one CA is defined as the number of live births or stillbirths having a specified CA that was identified from the DAD, expressed as a proportion of the total number of live births and stillbirths delivered in an Ontario hospital with infant residence in Ontario in the DAD.

Percent agreement and Kappa statistics were performed to assess the reliability (agreement) of CAs identified in the linked BIS and CIHI-DAD birth records. We applied the following conventional criteria to judge the strength of the agreement: Kappa coefficient <0: less than chance agreement; 0.01–0.20: slight agreement; 0.21–0.40: fair agreement; 0.41–0.60: moderate agreement; 0.61–0.80: substantial agreement; 0.81–0.99: almost perfect agreement (12, 13). For those anomalies with Kappa coefficients <0.9, we examined potential reasons for these discrepancies. All data linkages and analysis were performed using SAS 9.4.

### Ethics Consideration

As a quality assurance project, this data quality assessment was exempt from Research Ethics Board review under article 2.5 of the TCPS2 (the overarching ethical framework for research involving human participants in Canada) (14).

## Results

**Figure 1:**
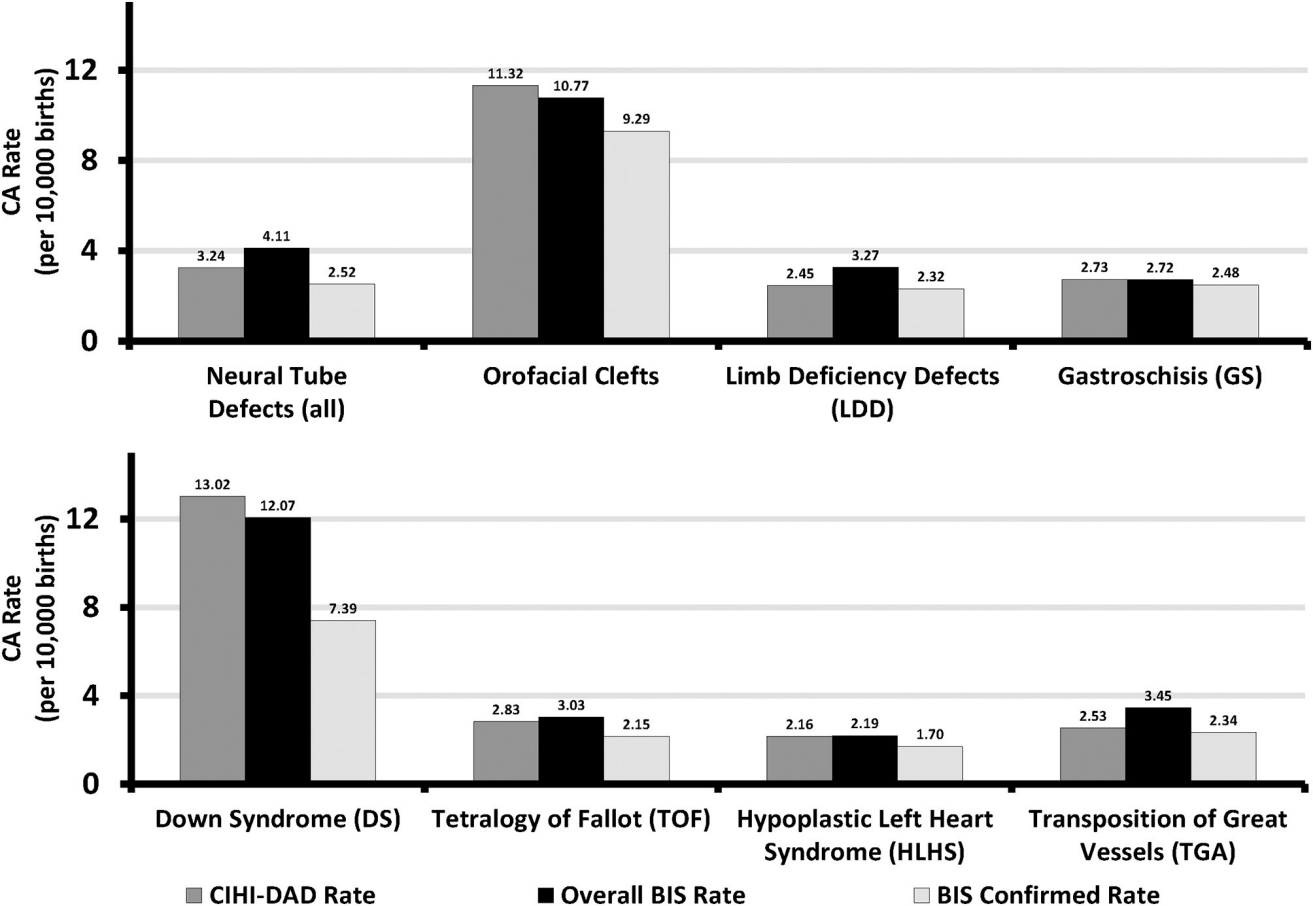
Sentinel Anomalies Prevalence Rates in Ontario, Canada (March 31 2012 - April 1 2013 to March 31 2015 - April 1 2016). BIS, BORN Information System; CIHI, Canadian Institute for Health Information; DAD, Discharge Abstract Database.

Figure 1 shows eight CA prevalence rates captured by the BIS overall (“Confirmed” or “Suspected”), BIS “Confirmed” only, and CIHI-DAD from April 1 2012 to March 31 2016 including 4.11, 2.52, and 3.24 per 10,000 births for NTD, 10.77, 9.29, 11.32 per 10,000 births for orofacial clefts, 3.27, 2.32, 2.45 per 10,000 births for LDD, 2.72, 2.48, and 2.73 per 10,000 births for GS, 12.07, 7.39, 13.02 per 10,000 births for DS, 3.03, 2.15, 2.83 per 10,000 births for TOF, 2.19, 1.70, and 2.16 per 10,000 births for HLHS, and 3.45, 2.34, 2.53 per 10,000 births for TGA.

**Figure 2:**
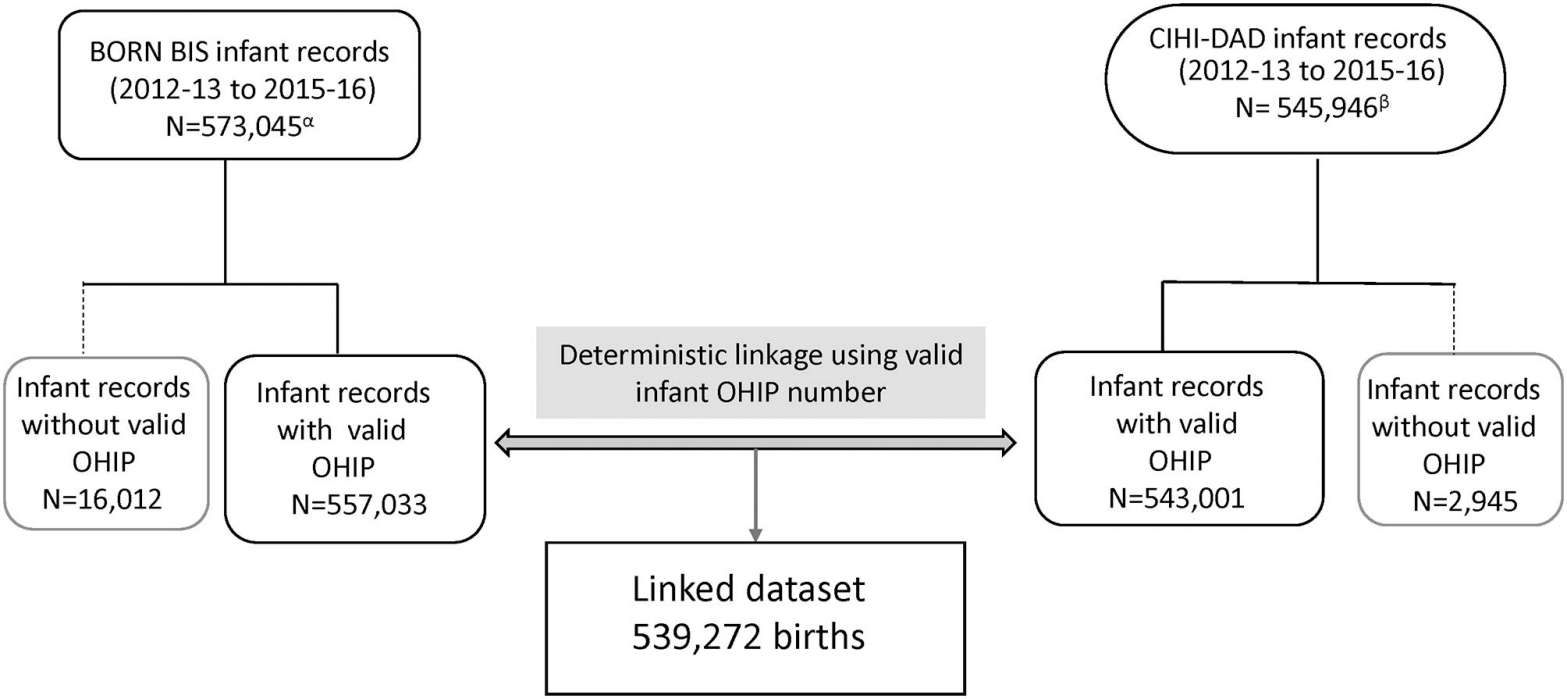
Flowchart of linkage for agreement evaluation in Ontario, Canada (March 31 2012 - April 1 2013 to March 31 2015 - April 1 2016). BIS, BORN Information System; CIHI, Canadian Institute for Health Information; DAD, Discharge Abstract Database; OHIP, Ontario Health Insurance plan. ^α^BORN BIS infant records include all birth records in Ontario. ^β^CIHI-DAD infant records include hospital births and infant mother's residence in Ontario only.

Over the study period, a total of 539,272 Ontario hospital birth records from the BIS and CIHI-DAD were linked using the infant's OHIP number. The linkage rates between the two databases were 96.8% (539,272 out of 557,033) in the BIS and 99.3% (539,272 out of 543,001) in the DAD (Figure 2). The total number of births from the BIS is higher than those from the DAD because the BIS also includes records of infants whose mothers delivery in Ontario but live outside of Ontario and records of infants are delivered outside of hospitals such as homes and birth centers. The DAD that we receive for analysis only includes records of infants whose mothers delivery babies in Ontario and live in Ontario, and the records of infants who are delivered in hospitals. Table 2 shows the number of CAs identified from the BIS and the DAD. Although percent agreements of all eight anomalies were over 99.9%, Kappa tests indicate that the degrees of agreement on diagnosis between BIS (“Confirmed” or “Suspected” cases) and CIHI-DAD were varied. The Kappa coefficients (κ) for GS, Orofacial clefts, DS, TOF, TGA, HLHS, NTD-all, and LDD were 0.96, 0.81, 0.75, 0.71, 0.62, 0.59, 0.53, and 0.30, respectively (Table 2). Except for the Kappa coefficient on GS (*K* = 0.96), the other seven anomalies had varied degrees of discrepancies. We thus further investigated the CA cases in the linked cohort that were captured in the DAD only (Table 3). All births were recorded as live births in both the BIS and DAD and there were only a small number of infants/fetuses whose GA at birth were <30 weeks (<5%). The neonatal transfer rate ranged from 28.36 to 76.03% among the records where CAs were identified in DAD birth records only. Regarding the most frequent and second most frequent ICD-10-CA codes of these seven anomalies identified in the DAD but not the BIS, we found Q05.9 and Q05.8 for NTD; Q35.9 and Q36 for orofacial clefts; Q71.3 and Q72.8 for LDD; Q90.9 and the number of other ICD-10-CA code <6 for DS; Q20.1 and Q20.38 for TGA. There were no subtypes of ICD-10-CA for TOF and HLHS.

**Table 2:** Reliability Assessment on Selected Congenital Anomalies between CIHI-DAD and BIS Birth Records (Ontario, 2012-2013 to 2015-2016)^*^.

**Selected sentinel congenital anomalies**	BIS (N)^*^	DAD (N)^*^	**Percent agreement**	**Kappa coefficient**	**Kappa 95% CI**		**Kappa *p*-value**
					**Lower limit**	**Upper limit**	
Neural tube defects (NTD)	158	129	99.97	0.53	0.46	0.60	<0.0001
Orofacial clefts	557	600	99.96	0.81	0.78	0.83	<0.0001
Limb deficiency defects (LDD)	152	124	99.96	0.30	0.23	0.37	<0.0001
Gastroschisis (GS)	140	134	100.00	0.96	0.93	0.98	<0.0001
Down syndrome (DS)	591	637	99.94	0.75	0.72	0.77	<0.0001
Tetralogy of Fallot (TOF)	151	136	99.98	0.71	0.65	0.77	<0.0001
Hypoplastic left heart syndrome (HLHS)	85	72	99.99	0.59	0.49	0.68	<0.0001
Transposition of great vessels (TGA)	166	117	99.98	0.62	0.55	0.68	<0.0001

* *Data was linked between BIS and CIHI-DAD using the baby's valid OHIP number. Most stillbirths were excluded due to lack of the valid OHIP number. In BIS, both “Confirmed” and “Suspected” cases were included. In addition, CA cases were identified from newborn anomalies for both singletons and high-order multiple births and fetal anomalies for singletons only. BIS, BORN Information System; CIHI, Canadian Institute for Health Information; DAD, Discharge Abstract Database*.

**Table 3:** Distribution of Selected Congenital Anomalies Identified in CIHI-DAD only (Ontario, 2012-2013 to 2015-2016)^¥^.

**Selected sentinel congenital anomalies**	**Cases captured in DAD only, N**	Gestational age at birth <30 weeks, N (%)^*^	Neonatal transfer, N (%)^*^	**Most frequent ICD-10-CA Q code in DAD, N (%)**	**Second most frequent ICD-10-CA Code in DAD, N (%)**	**List of all other ICD-10-CA code in DAD**
Neural tube defects (NTD)	53	<6	16 (30.19%)	Q05.9 (NTD unspecified), *N* = 28 (58.23%)	Q05.8 (Sacral spina bifida without hydrocephalus), *N* = 7 (13.21%)	Q00.0, Q01.1, Q01.2, Q01.8, Q01.9, Q04.8, Q05.4, Q05.6, Q05.7
Orofacial clefts	134	6 (4.48%)	38 (28.36%)	Q35.9 (Cleft palate, unspecified), *N* = 49 (36.57%)	Q36 (Cleft lip), *N* = 32 (23.88%)	Q35.1, Q35.3, Q35.5, Q37
Limb deficiency defects (LDD)	83	<6	26 (31.33%)	Q71.3 [Congenital absence of hand and finger(s)], *N* = 21 (25.30%)	Q72.8 [Other reduction defects of lower limb(s)], *N* = 20 (24.10%)	Q71.2, Q71.4, Q71.6, Q71.8, Q71.9, Q72.3, Q72.4, Q72.9, Q73.0, Q73.8
Down syndrome (DS)	179	7 (3.91)	63 (35.20%)	Q90.9 (Down syndrome, unspecified), *N* = 173 (96.65%)	NA	Q90.1, Q90.2
Tetralogy of Fallot (TOF)	34	<6	21 (61.76%)	No sub-group Q code	NA	NA
Hypoplastic left heart syndrome (HLHS)	26	<6	19 (76.03%)	No sub-group Q code	NA	NA
Transposition of great vessels (TGA)	30	<6	13	Q20.1 (Double outlet right ventricle), *N* = 12 (40.00%)	Q20.38 (Other transposition of great vessels NEC), *N* = 8 (26.67%)	Q20.30, Q20.31, Q20.58

¥ *The selected anomalies identified in CIHI-DAD only were all live births. The number of gastroschisis cases identified only in DAD birth records is <6, so gastroschisis cannot be shown in the above table in order to conform with privacy restrictions. Similarly, all numbers <6 in each cell are not shown. All % represents the percent of all cases identified in each anomaly. The most and second most frequent ICD-10-CA codes are not mutually exclusive. Due to privacy restrictions, only ICD-10-CA codes are listed in the last column. Coding manual can be accessed through the following link: https://secure.cihi.ca/free_products/CodingStandards_v2018_EN.pdf*.

* *Based on information from the BIS. Missing values were excluded to calculate rate (%). % of neonatal transfers was not reported for TGA due to missing values >30%*.

*BIS, BORN Information System; CIHI, Canadian Institute for Health Information; DAD, Discharge Abstract Database; NA, not applicable; GA, gestational age*.

## Discussion

In this study we calculated three types of prevalence rates for eight selected anomalies: overall prevalence (suspected + confirmed) in the BIS, confirmed prevalence in the BIS, and prevalence in the DAD. The prevalence rates of these anomalies have varied in the international literature. Certain CA rates identified in the BIS and CIHI-DAD over the study period are comparable with the reported CA rates in other countires (2). The average prevalence of GS and TOF were estimated as 2.75 per 10,000 births in the years of 2014-2015 and 2.82 per 10,000 births in the years of 2003-2012, respectively, in the European Union countries; the overall BIS and CIHI-DAD rates were almost the same (15, 16). Furthermore, the prevalence rates of DS, HLHS, TGA and orofacial clefts were in the range of that reported in the province of Alberta Canada, certain states of the USA and European countries (15–18). The lowest rates of NTD (6.3 per 10,000 births) and LDD (3.3 per 10,000 births) in the literature were still higher than those reported in Ontario, which suggests that both the BIS and DAD may not capture all cases (15, 16, 18).

The prevalence rates for anomalies identified from the BIS (overall or confirmed) and DAD in the 4-year period varied by type of anomaly. These three types of prevalence rates were almost identical for GS. The prevalence rate for orofacial clefts and DS were higher in the DAD than the overall and confirmed prevalence rates in the BIS. Furthermore, for LDD and TGA the prevalence rate in the DAD was lower than the overall BIS rate but close to the confirmed BIS rate. Lastly, for NTD and HLHS the prevalence rate in the DAD was lower than the overall BIS rate but higher than the confirmed BIS rate.

Similarly, the degree of agreement varied on different CAs captured in the BIS and CIHI-DAD. The highest degree of agreement was gastroschisis (κ = 0.96, close to one), suggesting almost perfect agreement on GS between the two databases. The next highest Kappa coefficient was oral-facial clefts (κ = 0.81) falling into the almost perfect category as well. DS (κ = 0.75), TOF (κ = 0.71), and TGA (κ = 0.62) fell into the substantial agreement category. HLHs (κ = 0.59) and NTD (κ = 0.53) were in the moderate agreement category. The lowest agreement was LDD (κ = 0.30) which indicated slight agreement.

Both crude comparisons of prevalence rates and Kappa test show that there is a high concordance on GS case ascertainments in the BIS and CIHI-DAD. GS is a birth defect occurring in the early stage of fetal development and is commonly diagnosed at 18–20 weeks of pregnancy by an ultrasound test. The diagnosis is usually easily confirmed after birth when the infant's intestines or other organs are seen outside of the infant's body (19). No additional tests are necessary for diagnosis, it is only very rarely confused with a ruptured omphalocele (Q79.2) and again the diagnosis is made on clinical examination. Thus, compared to most other anomalies, GS is easier to achieve a higher agreement on case ascertainment between clinical workers who enter the clinical information in the BIS and CIHI-DAD.

For the remaining seven anomalies, several factors may explain the discrepancies of anomaly capture between the two databases. First, compared to healthy newborns, infants with anomalies were likely transferred from a labor and birth department to a NICU or SCN for specialized care. Currently, the BIS only captures records in four of eight Ontario level III NICU sites. Therefore, the BIS may not be capturing the anomalies if the diagnosis was completed at a SCN or NICU. Our investigation results in Table 3 support this hypothesis. The overall neonatal transfer rate was around 10% in this linked cohort; however, a higher neonatal transfer rate to a SCN or NICU rate (28.36–76.03%) was found among the records where CAs were identified only in DAD birth records (Table 3).

Second, BORN's CA picklist values, representing specific anomalies, were developed and enhanced by clinical experts. There were around six hundred picklist values (diagnoses) in the BIS. However, the BORN picklist values may not cover all anomalies such as minor and rare cases. Conversely, CIHI-DAD uses the ICD-10-CA classification to classify anomalies. Anomalies are coded in the categories of Q00-Q99. Each anomaly or anomaly sub-category code starts with a letter “Q,” followed by two or three digit numbers. The letter “Q” represents “congenital anomalies” and the next two or three digits represent specific anomalies or a group of anomalies. Since BORN and ICD-10-CA are two different coding systems, there is a discrepancy between clinical diagnoses and the ICD-10-CA classification for certain sub-types of CA, posing a challenge for matching. In this linked cohort, the lowest Kappa value (k = 0.30) of LDD suggests most cases captured in the BIS and DAD are different infant records (55% of 152 LDD in BIS and 67% of 124 LDD in DAD). We further compared the BIS picklist values and ICD-10-CA categories for LDD and found there are some discrepancies for diagnoses or classification. In the DAD, the ICD-10-CA classifies LDD into three broader categories. They are Q71 for “Reduction defects of upper limb”; Q72 for “Reduction defects of lower limb”; and Q73 for “Reduction defects of unspecified limb.” On the other hand, in the BIS picklist, two general picklist values of “Generalized/other-Limb reduction defect(s) (LRD) - upper limb” and “Generalized/other-Limb reduction defect(s) (LRD) - lower limb” are able to match all subcategories under Q71 and Q72, respectively, in the ICD-10-CA. Specific LDD including “Clenched hands (persistently)” and “Radial ray anomaly (absent thumb)” can be grouped into the ICD-10-CA code Q71.3. The BIS picklist values of “Ectrodactyly (lobster-claw/cleft hand)” and “Generalized/other-Phocomelia” are matched with Q71.6 and Q73.1, respectively. However, the single picklist value of “Adactyly (absent fingers/ toes)” cannot be matched with neither Q71 nor Q72. In addition, there is one specific picklist value of “Generalized/other-Phocomelia” in the BIS to match a specific Q73.1. In addition, the BIS only contains one pick list value that aligns with sub-category Q73.1 but does not contain any pick list values that align with sub-categories Q73.0, Q73.8, or the overarching category Q73. Furthermore, we also found the most frequent ICD-CA Q for LDD is Q71.3 [Congenital absence of hand and finger(s), *N* = 21 (25.30% of 83)] and the second most frequent ICD-10-CA Code in DAD is Q72.8 [Other reduction defects of lower limb(s), *N* = 20 (24.10% of 83)]. Generally speaking, compared with other more severe anomalies, absence of one finger, one toe or not otherwise specified (NOS) reduction defects of lower limb(s) is much milder and less noticeable. Therefore, it is highly unlikely to be captured in both the BIS and DAD (personal communications with clinical experts).

Finally, CA data entry only captures a “snapshot” of CA diagnosis in both the BIS and DAD (personal communication with clinical experts in Ontario). Thus, there is a potential that anomaly diagnosis was corrected after further clinical investigation. However, we lack “follow-up” data to correct the diagnosis. It is possible that there is a misclassification of CA diagnosis in both databases including DS, NTD, TOF, HLHS and TGA, which require additional investigations including genetic testing, neuroimaging or echocardiography, for accurate diagnosis and anomaly classification. Table 3 shows Q05.9, Q35.9 and Q90.9 are the most frequent ICD-10-CA captured in the DAD but not in the BIS. These codes are all in the “unspecified” categories implying that more specified CA diagnosis may be needed after receiving test results (personal communications with clinical experts during the PHAC annual meeting).

This study has several limitations. First, we assessed the agreement of selected anomalies between two databases and discussed that the way this information is captured in the BIS may contribute to discrepancies. Alternatively, anomaly case ascertainment discrepancies could also be due to how the DAD data are collected, which we are unable to evaluate. In the current study, we were not able to ensure which diagnosis represents the truth; in future work we plan to conduct a chart review to verify the discrepancies in diagnoses between two databases. A re-abstraction study may also be needed to evaluate the validity of anomaly data entry in the BIS. Second, in cases of multiple gestation, there is a challenge with the reliable identification of fetus A/B/C etc. throughout the pregnancy (across scans and across antenatal specialty/prenatal screening follow-up encounters), and subsequent challenges with accurately linking each fetus to the respective infant in the BIS infant datasets. In order to minimize misclassification bias, we only linked fetal anomalies in singletons and all newborn anomalies in the BIS databases. This limitation led to fewer fetal anomalies identified in the BIS. However, the proportion of fetal anomalies (<5%) in multiple gestation pregnancies is small in the BIS prenatal databases. Third, the discrepancies between the two databases on the selected CA diagnoses could be due to a potential linkage error, which may occur when the data entry personnel manually enter infants' OHIP numbers (20). However, in our study we expect the impact of this type of linkage error is minor because both BORN Ontario and CIHI apply strict rules and algorithms to identify correct infant OHIP numbers (5, 21). Finally, unlike the active surveillance system in the province of Alberta, Canada (22), a passive case ascertainment method is being adopted for the Ontario congenital anomaly surveillance. Cases are identified from different data sources. This method is the most feasible and cost efficient method for current surveillance in Ontario. Case reports and data entries are voluntary, which might contribute to issues such as incomplete data entry and inaccurate case ascertainment (23, 24). BORN has strived to develop strategies to improve case ascertainment and enhance the CA surveillance data quality.

## Conclusions

In summary, we found a high percent agreement between the BIS and CIHI for all anomalies in this study, although the degree of agreement varied among sentinel CAs identified. The potential reasons for discrepancies include incompleteness of capturing CAs using existing picklists, especially for certain sub-types, incomplete NICU data in the BIS, and differences between clinical diagnosis in the BIS and ICD-10-CA coding in the CIHI-DAD. In order to increase the accuracy of anomalies ascertainment and enhance the capture of anomaly data, BORN has developed strategies to find more data sources including recruiting more NICU/SCNsites to contribute newborn anomaly data, developing a new database to improve the completeness of fetal anomaly data entry, and linking cytogenetics data for verification of chromosomal anomalies ascertainment. BORN is collaborating with additional data partners that can contribute to greater ascertainment of CAs.

The next step will be a data abstraction study to explore and investigate the potential reasons for discrepancies based on hospital chart review. This project helps quantify the quality of CA data collection in the BIS, enhances understanding of CA prevalence in Ontario and provides direction for future data quality improvement activities.

## Data Availability Statement

The datasets generated and analyzed during the current study is held securely at the prescribed registry BORN Ontario. Data sharing regulations prevent this data from being made available publicly due to the personal health information in the datasets. Enquiries regarding BORN data must be directed to BORN Ontario (Science@BORNOntario.ca).

## Ethics Statement

Ethical review and approval was not required for the study on human participants in accordance with the local legislation and institutional requirements. Written informed consent for participation was not provided by the participants' legal guardians/next of kin because as a quality assurance project, this data quality assessment was exempt from Research Ethics Board review in Canada (14).

## Author Contributions

QM: project development, data management and analysis, manuscript writing, and editing. SD and AM: project development, manuscript writing, and editing. All authors have read and approved the manuscript.

## Conflict of Interest

The authors declare that the research was conducted in the absence of any commercial or financial relationships that could be construed as a potential conflict of interest.

## Abbreviations

BIS: The BORN Information System (BIS)
CAs: congenital anomalies
CIHI: the Canadian Institute for Health Information
DAD: Discharge Abstract Database
DS: Down syndrome
HLHS: hypoplastic left heart syndrome
GS: gastroschisis
LDD: limb deficiency defects
NTD: neural tube defects
TGA: transposition of great vessels
TOF: tetralogy of Fallot.
